# Supplementary material for: Qualitative description of interpersonal HIV stigma and motivations for HIV testing among gays, bisexuals, and men who have sex with men in Ghana’s slums—BSGH-005
Source: PLoS One. 2024 Jun 5;19(6):e0289905. doi: 10.1371/journal.pone.0289905 (PMC11152275; doi:10.1371/journal.pone.0289905)
Supplement: Attachment — Submitted filename: PONE-D-23-23534_Reviewer.pdf [file pone.0289905.s002.pdf]

PLOS ONE

Qualitative description of interpersonal HIV stigma and motivations for HIV testing among gays, bisexuals, and men who have men in Ghana's slums- BSGH-005

--Manuscript Draft--

**Manuscript Number:**	PONE-D-23-23534
**Article Type:**	Research Article
**Full Title:**	Qualitative description of interpersonal HIV stigma and motivations for HIV testing among gays, bisexuals, and men who have men in Ghana's slums- BSGH-005
**Short Title:**	HIV stigma and motivations for testing among GBMSM.
**Corresponding Author:**	Osman Wumpini Shamrock, Ph.D<br>University of Rochester School of Nursing<br>Rochester, New York UNITED STATES
**Keywords:**	HIV testing, interpersonal HIV stigma, HIV testing motivations, GBMSM, Slums, Ghana
**Abstract:**	Abstract<br>Despite significant progress in Ghana's HIV response, disparities in HIV prevalence persist among different populations. Gays, bisexuals, and other men who have sex with men (GBMSM) in the country remain vulnerable to HIV infection due to high levels of stigma and discrimination, limited access to healthcare services, and low HIV knowledge levels. While limited studies focus on HIV prevention and care in the Ghanaian GBMSM context, we did not find studies on GBMSM in slums. We, therefore, explored stigma and motivations of HIV testing among GBMSM in slums. In collaboration with our community partners, we recruited and conducted face-to-face interviews among 12 GBMSM from slums in Accra and Kumasi, Ghana. Our multiple-reviewer summative content analysis identified the following: under HIV stigma, we identified two categories, avoidance of GBMSM living with HIV and fear of testing positive for HIV. Under motivations for HIV testing, we identified three categories; HIV vulnerability, sexual health decision making, and positive messaging about HIV. Our findings provide valuable insights into stigma and motivations for HIV testing among GBMSM in Ghanaian slums. They also highlight the importance of targeted HIV education interventions to empower GBMSM to take responsibility for their sexual health and address the unique challenges they face accessing HIV testing services.
**Order of Authors:**	Gamji Rabiu Abu-Ba’are, Ph.D
	Edem Yaw Zigah, BA
	Osman Wumpini Shamrock, Ph.D
	Adedotun Ogunbajo, Ph.D
	Henry Delali Dakpui, MSc
	George Rudolph Kofi Agbemedu, BA
	Donte T Boyd, Ph.D
	Oliver C Ezechi, Ph.D
	LaRon E Nelson, Ph.D
	Kwasi Torpey, Ph.D
**Additional Information:**	
**Question**	**Response**
**Financial Disclosure**<br><br>Enter a financial disclosure statement that describes the sources of funding for the work included in this submission. Review	GRA applied and received grant funding from the Yale university FLAGS grant. Funding agency did not play a role in the study design, data collection, and analysis, decision to publish or preparation of the manuscript. All content is solely those of the authors and does not represent that of the funding agency.

Powered by Editorial Manager® and ProduXion Manager® from Aries Systems Corporation

the submission guidelines for detailed requirements. View published research articles from *PLOS ONE* for specific examples.<br><br>This statement is required for submission and **will appear in the published article** if the submission is accepted. Please make sure it is accurate.<br><br>**Unfunded studies**<br>Enter: *The author(s) received no specific funding for this work.*<br><br>**Funded studies**<br>Enter a statement with the following details:<br>• Initials of the authors who received each award<br>• Grant numbers awarded to each author<br>• The full name of each funder<br>• URL of each funder website<br>• Did the sponsors or funders play any role in the study design, data collection and analysis, decision to publish, or preparation of the manuscript?<br>• **NO** - Include this sentence at the end of your statement: *The funders had no role in study design, data collection and analysis, decision to publish, or preparation of the manuscript.*<br>• **YES** - Specify the role(s) played.<br><br>* typeset	
**Competing Interests**<br><br>Use the instructions below to enter a competing interest statement for this submission. On behalf of all authors, disclose any competing interests that could be perceived to bias this work—acknowledging all financial support and any other relevant financial or non-financial competing interests.<br><br>This statement is required for submission and **will appear in the published article** if the submission is accepted. Please make sure it is accurate and that any funding sources listed in your Funding Information later in the submission form are also	The authors have declared that no competing interests exist.

declared in your Financial Disclosure statement.<br><br>View published research articles from *PLOS ONE* for specific examples.<br><br>**NO authors have competing interests**<br><br>Enter: *The authors have declared that no competing interests exist.*<br><br>**Authors with competing interests**<br><br>Enter competing interest details beginning with this statement:<br><br>*I have read the journal's policy and the authors of this manuscript have the following competing interests: [insert competing interests here]*<br><br>* typeset	
**Ethics Statement**<br><br>Enter an ethics statement for this submission. This statement is required if the study involved:<br><br>• Human participants<br>• Human specimens or tissue<br>• Vertebrate animals or cephalopods<br>• Vertebrate embryos or tissues<br>• Field research<br><br>Write "N/A" if the submission does not require an ethics statement.<br><br>General guidance is provided below. Consult the submission guidelines for detailed instructions. **Make sure that all information entered here is included in the Methods section of the manuscript.**	Ethical approval was received from the Ghana Health Service Ethics Committee (GHS-ERC 011/10/21) and the Institutional Review Board Committee (IRES IRB) of Yale University (IRES IRB #RNI2000030856). Written consent was collected from all participants.

Powered by Editorial Manager® and ProduXion Manager® from Aries Systems Corporation

**Format for specific study types**<br><br>**Human Subject Research (involving human participants and/or tissue)**<br>• Give the name of the institutional review board or ethics committee that approved the study<br>• Include the approval number and/or a statement indicating approval of this research<br>• Indicate the form of consent obtained (written/oral) or the reason that consent was not obtained (e.g. the data were analyzed anonymously)<br><br>**Animal Research (involving vertebrate animals, embryos or tissues)**<br>• Provide the name of the Institutional Animal Care and Use Committee (IACUC) or other relevant ethics board that reviewed the study protocol, and indicate whether they approved this research or granted a formal waiver of ethical approval<br>• Include an approval number if one was obtained<br>• If the study involved *non-human primates*, add *additional details* about animal welfare and steps taken to ameliorate suffering<br>• If anesthesia, euthanasia, or any kind of animal sacrifice is part of the study, include briefly which substances and/or methods were applied<br><br>**Field Research**<br><br>Include the following details if this study involves the collection of plant, animal, or other materials from a natural setting:<br>• Field permit number<br>• Name of the institution or relevant body that granted permission	
**Data Availability**<br><br>Authors are required to make all data underlying the findings described fully available, without restriction, and from the time of publication. PLOS allows rare exceptions to address legal and ethical concerns. See the PLOS Data Policy and FAQ for detailed information.	Yes - all data are fully available without restriction

A Data Availability Statement describing where the data can be found is required at submission. Your answers to this question constitute the Data Availability Statement and **will be published in the article**, if accepted.<br><br>**Important:** Stating 'data available on request from the author' is not sufficient. If your data are only available upon request, select 'No' for the first question and explain your exceptional situation in the text box.<br><br>Do the authors confirm that all data underlying the findings described in their manuscript are fully available without restriction?	
**Describe where the data may be found in full sentences. If you are copying our sample text, replace any instances of XXX with the appropriate details.**<br><br>• If the data are **held or will be held in a public repository**, include URLs, accession numbers or DOIs. If this information will only be available after acceptance, indicate this by ticking the box below. For example: *All XXX files are available from the XXX database (accession number(s) XXX, XXX.*).<br>• If the data are all contained **within the manuscript and/or Supporting Information files**, enter the following: *All relevant data are within the manuscript and its Supporting Information files.*<br>• If neither of these applies but you are able to provide **details of access elsewhere**, with or without limitations, please do so. For example:<br><br>*Data cannot be shared publicly because of [XXX]. Data are available from the XXX Institutional Data Access / Ethics Committee (contact via XXX) for researchers who meet the criteria for access to confidential data.*<br><br>*The data underlying the results presented in the study are available from (include the name of the third party*	All data contain in the study can be found in the manuscript.

and contact information or URL).<br>• This text is appropriate if the data are owned by a third party and authors do not have permission to share the data.<br><br>* typeset	
Additional data availability information:	

Powered by Editorial Manager® and ProduXion Manager® from Aries Systems Corporation

Manuscript

Click here to access/download;Manuscript;Stigma and Testing Motivations.docx

Qualitative description of interpersonal HIV stigma and motivations for HIV testing among gays, bisexuals, and men who have sex with men in Ghana's slums - BSGH-005

Gamji Rabiu Abu-Ba'are*[1,2,3,4,7.9], Edem Yaw Zigah*[2], Osman Wumpini Shamrock[1,2,3], Adedotun Ogunbajo[6], Henry Delali Dakpui[2], George Rudolph Kofi Agbemedu[2], Donte Boyd[7], Oliver Ezechie[8], LaRon Nelson [5,9], Kwasi Torpey[10]

1. Behavioral, Sexual, and Global Health Lab, School of Nursing, University of Rochester, Rochester, New York, USA.
2. Behavioral, Sexual, and Global Health Lab, Jama'a Action, West Legon, Accra, Ghana.
3. School of Nursing, University of Rochester, Rochester, New York, USA.
4. Department of Public Health Sciences, University of Rochester, Rochester, New York
5. School of Nursing, Yale University, New Haven, Connecticut, USA.
6. Us Helping Us People into Living, Washinton, DC, USA
7. College of Social Work, Ohio State University, Columbus, USA
8. Clinical Sciences Department, Nigerian Institute of Medical Research, Lagos, Nigeria.
9. Center for Interdisciplinary Research on AIDS, School of Public Health, Yale University
10. School of Public Health, University of Ghana

Abstract

Despite significant progress in Ghana's HIV response, disparities in HIV prevalence persist among different populations. Gays, bisexuals, and other men who have sex with men (GBMSM) in the country remain vulnerable to HIV infection due to high levels of stigma and discrimination, limited access to healthcare services, and low HIV knowledge levels. While limited studies focus on HIV prevention and care in the Ghanaian GBMSM context, we did not find studies on GBMSM in slums. We, therefore, explored stigma and motivations of HIV testing among GBMSM in slums. In collaboration with our community partners, we recruited and conducted face-to-face interviews among 12 GBMSM from slums in Accra and Kumasi, Ghana. Our multiple-reviewer summative content analysis identified the following: under HIV stigma, we identified two categories, avoidance of GBMSM living with HIV and fear of testing positive for HIV. Under motivations for HIV testing, we identified three categories; HIV vulnerability, sexual health decision making, and positive messaging about HIV. Our findings provide valuable insights into stigma and motivations for HIV testing among GBMSM in Ghanaian slums. They also highlight the importance of targeted HIV education interventions to empower GBMSM to take responsibility for their sexual health and address the unique challenges they face accessing HIV testing services.

Keywords: HIV testing, interpersonal HIV stigma, HIV testing motivations, GBMSM, Slums, Ghana

Sub-Saharan Africa (SSA) carries over two-thirds of the world's burden of HIV, yet, HIV testing and related services remain underutilized due to several factors such as insufficient knowledge, low-risk perception, and increased stigma. (1,2) Despite programmatic efforts, the burden of HIV and the factors (e.g., stigma) that hinder access to HIV testing remain a significant obstacle to HIV testing among HIV key populations such as gays, bisexuals, and men who have sex with men (GBMSM) (3–5)

Although Ghana has made significant strides in its HIV response, disparities persist in HIV prevalence between different populations, with GBMSM carrying a disproportionate burden, 18% compared to the nations, 1.7%% (6–8). Efforts to increase HIV testing and prevention among GBMSM continue, yet reports indicate suboptimal testing rates among GBMSM in the country due to individual and environmental barriers (6,7,9,10). At the personal level, such factors include low-risk perception, low HIV knowledge, and fear of rejection (3,11). At the environmental level, factors include healthcare facility-level stigma, inadequate access to testing, and community stigma among others(11–14). At the peer level, interpersonal HIV stigma affects interest in testing as some GBMSM discriminate against their peers living with HIV.(3,11)

While limited studies explore stigma and testing practices among GBMSM in Ghana, no known studies have focused explicitly on GBMSM in Ghanaian slum communities. However, studies in low-income settings identify association between low HIV knowledge and low HIV testing among persons with low socioeconomic status(15,16). Slums also remain associated with high-risk behaviors such as transactional sex, inconsistent condom use, and increased HIV prevalence (17,18). Emerging findings from eastern Africa show that GBMSM in slums have low-risk perception, increased risk behaviors, and low access to HIV testing and prevention services (19,20). For instance, in Kenya, HIV rate among slum residents, 12% was higher than 5% and 6%

among non-slum urban and rural residents, respectively (20). Also, out of 4028 youth sampled in Kenyan slums, only 27% had ever tested for HIV, and over 90% had low HIV risk perception. Of those who tested, over 90% reported being required to take the test (19).

Whereas no specific studies exist among GBMSM in Ghana's slums, previous studies among slum communities in Ghana report increased HIV risk behaviors and higher HIV prevalence among slum residents. These communities also increased HIV prevalence and risk – have poorly resourced infrastructure and health facilities leading to adverse health outcomes (21,22). Urban areas such as the Accra and Kumasi larger regions, where GBMSM were sampled for the study, present the highest prevalence rates of HIV, 2.47% and 1.98%, respectively, compared to the other 16 regions (8). These two cities and their surrounding areas also record high prevalence among GBMSM, Accra (42%) and Kumasi (25%), compared to the national GBMSM rate of 17.5% (6). Thus, placing GBMSM living in urban slums at increased risk of HIV infection than other populations.

The current study seeks to understand GBMSM slum-specific HIV stigma and motivation for testing in Accra and Kumasi, Ghana. Understanding stigma and motivations for HIV testing is critical to reducing HIV transmission and improving health outcomes for this population. As the Self Determination Theory (SDT) explains, individuals' innate psychological needs inform their well-being and quality of life within a social context (23,24). The various components of SDT (the basic psychological need for autonomy, competence, and relatedness) could help explain the importance of HIV testing motivations among GBMSM in slums. Per the psychological need for autonomy, the barriers faced by GBMSM in slums can undermine the autonomy and restrict their ability to make informed HIV testing (11,12,25,26). On the psychological need for competence, the low levels of HIV knowledge can affect GBMSM's proficiency to make informed decisions

regarding HIV testing (25,27). Also, the stigma and discrimination faced by GBMSM can negatively impact the relatedness aspect of SDT, which emphasizes the need for individuals to feel connected and supported in their social environments. Creating supportive and inclusive environments, reducing stigma, and improving access to healthcare services are crucial for promoting relatedness and encouraging GBMSM to seek HIV testing (28–30).

Methodology

Sampling and Recruitment Procedure

Using the time location sampling (TLs) technique, we reached and recruited GBMSM in slum communities in collaboration with our community partners in Accra and Kumasi. Research assistants working with our community partner organizations in Accra (Priorities on Rights and Sexual Health - PORSH) and Kumasi (Youth Alliance on Health and Human Rights - YAHR) screened and invited GBMSM to take part in interviews sessions during one of the organizations' activities when GBMSM visited the site. We have worked with these community partners in previous studies (9,10). Although we originally intended to find 19 participants, after the eighth interview, we reached saturation and added 4 to ensure complete information saturation, making our total transcripts 12

Inclusion criteria

Participants in the study had to be at least 18 years old and live in a slum community in Accra, Ghana's Greater Accra regional capital, or Kumasi, Ghana's Ashanti regional capital. Additionally, the individual must identify as a cisgender man who self-identifies as gay, bisexual, or pansexual or engage in sexual intercourse with another cis-gender man for reasons other than

sexual orientation. The person must have been sexually active during engagement and had sexual intercourse with another cis-gender man within the previous six months.

Data Collection Procedure

Procedure

To gather information from the participants, we conducted in-depth face-to-face interviews. Following the screening, participants were given consent forms by the research assistants to review. The research assistants also read the consent forms out loud and provided extra explanations to ensure everything was understood. They answered the participants' queries before the interviews started, collected signatures confirming GBMSM's agreement to participate in the study, and allow for audio recording. The community partners' private spaces were used for all conversations. All but four interviews were in English, the other four in Twi, a local Ghanaian language that some participants found more conversant. Data were collected from participants from 01/21/2022 to 02/26/2022.

Nature of questions

The research assistants were trained to conduct qualitative interviews using the study's checklist as a guide in collecting information on HIV stigma and motivations for testing among GBMSM living in Ghanaian slum communities. In line with our design, the checklist allowed a more transparent and open discussion rather than the traditional question-and-answer interview structure. Participants were asked to share their experiences of HIV testing, their knowledge, and what motivated or affected their interest in testing.

Analytical Strategy

Trained research assistants deidentified the transcripts after translating the audio interview recordings verbatim. We performed a summative content analysis on the transcripts with multiple reviewers (10). Our team has successfully used this analytical method to comprehend crucial components in participant accounts (10). Each transcript received at least two reviewers. Each reviewer independently read the transcripts to identify the most crucial points made by the participants. They then reported these points in between 100 and 200 words. The principal authors reviewed each summary to find clusters and recorded the elements frequently appearing in transcripts and summaries in a data spreadsheet. We identified several clusters and classified them under categories that outlined participant experiences, perceptions, and motivations for HIV testing. Each area that was reported appeared in both peer reviewers' summaries.

Ethical Considerations

Ethical approval was received from the Ghana Health Service Ethics Committee (GHS-ERC 001/10/21) and the Institutional Review Board Committee (IRES IRB) of Yale University (IRES IRB #RNI2000030856). The interviewers in this study ensured that each participant had read and understood the informed consent form thoroughly before any data was collected, and afterward, they obtained written consent.

Results

Description of participants

The 12 participants identified as cis-gender men and had sexual intercourse within the previous six months with another cis-gender man. Six participants identified as Christians, four as Muslims, and two as both Muslims and Christians. Five participants accomplished tertiary education, and six concluded senior high school. A member didn't complete Junior High school.

Description of findings

We organized the results into two main groups; 1) HIV stigma and 2) Motivations for HIV testing. Under HIV stigma, we identified two categories, avoidance of GBMSM living with HIV and fear of testing positive for HIV. Under motivations for HIV testing, we identified three categories; HIV vulnerability, sexual health decision-making, and positive messaging about HIV.

HIV Stigma

Avoidance of GBMSM living with HIV: Our results show participants had fears and misinformation concerning PLHIV in the slums. Fears stemmed from the possibility of acquiring HIV through everyday activities such as bathing or eating with PLHIV, suggesting a lack of understanding of how HIV is transmitted. Some participants avoided their peers living with HIV or maintained cordial relationships without sexual relations.

> I'm scared of them. I will be scared to eat with the person infected with HIV. Bath with the person or even live in the same area with the person. Because I don't want to get close to the person, maybe because my thoughts are that the person may transfer it to me at any moment, so I will neglect the person. (GBMSM participant)

> They do much of sex, they are sex addicts. Because when you don't have sex, you won't get infected. And married people usually don't get infected in my community. (GBMSM participant)

> I wouldn't mind eating with the person because I was told you can only get infected through sex, deep kissing and sharing sharps. So I will treat the person like an ordinary person. Sex will be the only thing I wouldn't have with this person. I am nondiscriminatory; that's why my friends came to me and told me they were positive. (GBMSM participant)

Fear of testing positive for HIV: Some participants expressed experience testing for HIV. However, they also shared that they are usually scared of testing for HIV due to the fear that they may test positive. Indicating low knowledge of HIV and its treatment options.

> It's scary, I have tested for HIV, but I won't like to share my status now. Being in the house, I encourage myself to go and get tested. However, when I get there, it's scary. What if it comes out positive? But rather, I should be thinking the other way that it should be negative. (GBMSM Participant).

> It's very scary going for HIV testing, but when you encourage yourself to do the test, you realize it's not that scary but just like the normal tests that we do. But because of the mentality that if you test, you will come out positive and the ARV and other stuff, its scary. (GBMSM Participant).

Motivations for HIV testing among GBMSM.

HIV vulnerability: Some GBMSM were motivated to test for HIV due to their HIV high-risk awareness, as being a sexually active GBMSM poses a higher risk of getting HIV because of the increased HIV prevalence compared to the general population. Some GBMSM acknowledged that some risky sexual behaviors further increased their risk of acquiring HIV, hence, felt the need to get tested.

> Yes, there's the need to test for HIV…because men who have sex with men have a higher rate of getting HIV infections compared to men having sex with ladies. So, it's very good for any MSM to get tested. (GBMSM participant).

> It is very scary when I am going to test. But I feel it's very important for me to know my status to prevent me from getting sick. So, it's very important for me to test for HIV. I test for HIV sometimes because maybe I've had raw sex with someone, and I don't trust the person (GBMSM participant).

> I do test for HIV because it's an opportunistic infection. And you will not know when you might contract it, so I have to test for it to know my status…the sex doesn't always go as planned…and risky behaviors. I have had some infections some time ago. And now I prefer to be extra careful (GBMSM participant).

Sexual health decision making. GBMSM accounts show they are motivated to test for HIV because they want to know their HIV status to make informed decisions about their sexual

health. They find testing crucial in seeking appropriate medical care and treatment should they turn positive, thereby promoting long-term health and well-being.

> Of course, it is necessary to get tested because it will help you to know your status and decision-making. if I test positive for HIV, there are medications to help me live long. (GBMSM participants)

> It's important for me to test for HIV…because you have to know your status. Knowing your status allows you to know what to do. The more you keep on delaying, the more the thing (HIV) too gets worse. Because maybe you are HIV positive, you aren't on drugs, and if you delay, it might turn into something else (AIDS). And that can cause your death (GBMSM participant).

> I feel it's important to test for HIV because sometimes you feel like you've been exposed (to HIV). Sometimes after having sex, you feel as if you should go, and your body isn't feeling too well. These thoughts just run through my head, so I go and test because I'm not comfortable with that. So, the earlier you get tested, the better (GBMSM participant).

Positive messaging about HIV: Participants cited their knowledge about HIV and the education and sensitization they received about HIV as a motivator for HIV testing. GBMSM in the study recognized that HIV is an opportunistic infection that could affect anyone, and often without any noticeable symptoms until it has progressed to AIDS. As a result, many GBMSM participants emphasized the importance of knowing their HIV status to ensure early detection and access to treatment if needed.

> Lately, I have been hearing a lot about HIV and how it's spreading. I went and took the test. And I found out I'm negative It is always good to know your status. When I had admitted to college, I had my test before I started lectures. Even if you test positive there are treatment options for you and I learned if you take your medication well, you will achieve the undetectable stage. So, I believe is good to test for HIV (GBMSM Participant).

> I thought HIV was so deadly until I started reading about it. I learned that malaria is even more dangerous than HIV, so I think everyone should test and know their status. And I believe persons living with HIV have the right to life, and they shouldn't be stigmatized or discriminated against… I've learned that there are drugs they give to them when they have HIV. And when they give it to them and take it as prescribed, you will be ok and fine. (GBMSM participant)

> I was told that if I should test positive for HIV, there were treatment options and other medications which would prolong my life. So, I always test when it's time for me to test. (GBMSM participant)

Discussion

Despite increased stigma, HIV vulnerability and low HIV testing associated with GBMSM and residents of slums, limited studies examine HIV testing among GBMSM, especially in slums in Ghana (10,31–33). The present study qualitatively describes GBMSM level stigma and their motivations for HIV testing in Ghana's slums. Whereas some GBMSM demonstrated an understanding and acceptance of people living with HIV, others avoided their peers living with HIV and had fears of testing for HIV. The need to test was driven by factors such as HIV vulnerability, sexual health decision making and positive messaging about HIV-informed motivations for HIV Testing. Highlighting the need for interventions to increase HIV knowledge and leverage the motivation to improve HIV testing among GBMSM in slums (4,34–37).

Although this is one of the early studies to explore GBMSM level HIV stigma in slum communities, the findings remain consistent with literature reported among GBMSM and other populations in Ghana and other SSA (4,34–39). Consistent with previous findings, HIV stigma undermines HIV testing decisions among some participants as they fear testing for HIV due to the stigma associated with testing positive (9,38–40). Thus, it remains imperative for interventions to address HIV stigma to improve testing (9,40,41). Such interventions remain essential considering that the participants did not only have negative misconceptions about HIV, they expressed stigma towards their peers living with HIV by avoiding them, refusing them sex and labeling them as sexually promiscuous. Such labeling and avoidance of others living with HIV reflect our previous findings among other GBMSM in Ghana (9,26,34,40,42). Such behavior poses a significant

challenge for testing and the willingness of GBMSM who test positive to disclose their status and even adhere to care as they will not want to be isolated by their peers (42–44).

Despite the stigma shown by others, findings around motivations for HIV testing provide opportunities for leveraging motivated GBMSM in slums to improve HIV knowledge and testing among their peers (10,11,31). As shown in the results, some GBMSM understand the basics of HIV, especially around risk behaviors, and respond to such risk by testing for HIV, which remains consistent with previous research that shows that the awareness of increased vulnerability among GBMSM encouraged them to test to protect themselves and their partners (12,34,45). Consistent with the SDT and prior literature, the participants' responses also show that increased knowledge of HIV will increase their self-determination for testing. GBMSM did not only consider HIV testing as a path to knowing their HIV status but also as an essential means for knowledge acquisition about their health to enable them to take meaningful steps like seeking care and adopting behavior changes to ensure their well-being. Whereas no previous studies focused on GBMSM in slums, earlier studies among GBMSM in Ghana also showed that some GBMSM acknowledged the importance of HIV testing, as it enabled them to know their status and make important health sexual health behavioral choices (12,34).

To emphasize further, participants' accounts show that positive messaging about HIV, instead of negative messaging can encourage HIV testing. Others informing participants about how HIV is transmitted and treated, including stages such as undetectable status motivated them to test for HIV. Previous studies reported similar findings among GBMSM (9,38,40). In one of the studies on HIV health promotion, after attending a workshop where GBMSM peer groups discussed and learned about HIV, they observed an increase in HIV testing from 4% to 17% within a week post-intervention (9).

Taken together, the central takeaway from the findings on stigma and motivation for HIV testing lies in the importance of empowering GBMSM to take charge of their sexual health and make informed decisions about HIV testing, as this may help in reducing barriers to testing and promote self-efficacy. By enhancing their sense of competence and autonomy, tailored interventions can motivate GBMSM living in Ghanaian slums to overcome the barriers to HIV testing and take control of their sexual health. A popular intervention that could help reduce HIV stigma at the individual and interpersonal level is the Many Men Many Voices (3MV) (9,40,46). The intervention addresses stigma, HIV risk, transmission, testing, and treatment among GBMSM in Black communities. When adapted to Ghana, we found that GBMSM improved their understanding of HIV, formed community, and improved their HIV testing behaviors (40).

Despite the important findings, research needs to consider the study limitations when applying our participants' accounts. As a qualitative study, which recruited from two regions in Ghana, the findings may not apply to all GBMSM in slums across other West African regions. We therefore recommend using these results in conjunction with others from the region to draw conclusions. We also recommend using other research designs, like quantitative or mixed methods, to minimize data collection and analysis bias in future studies. Future studies could include GBMSM from other regions and consider targeting specific age groups, as this formative work did not have any specific age brackets and may not fully represent people in different age groups.

In conclusion, our findings contribute to the existing knowledge and provide insights for policymakers, healthcare providers, and researchers to develop effective strategies and programs aimed at reducing HIV disparities and improving HIV testing among GBMSM in slums. While stigma can undermine HIV testing, some GBMSM are highly motivated to test for HIV as such

positive messaging about HIV should be encouraged and leveraged to increase HIV self-testing among GBMSM in Ghana’s slums.

References

1. Poteat T, Ackerman B, Diouf D, Ceesay N, Mothopeng T, Odette KZ, et al. HIV prevalence and behavioral and psychosocial factors among transgender women and cisgender men who have sex with men in 8 African countries: A cross-sectional analysis. PLoS Med. 2017;14(11).
2. Ahouada C, Diabaté S, Mondor M, Hessou S, Guédou FA, Béhanzin L, et al. Acceptability of pre-exposure prophylaxis for HIV prevention: Facilitators, barriers and impact on sexual risk behaviors among men who have sex with men in Benin. BMC Public Health. 2020;20(1).
3. Abubakari GM, Dada D, Nur J, Turner D, Otchere A, Tanis L, et al. Intersectional stigma and its impact on HIV prevention and care among MSM and WSW in sub-Saharan African countries: a protocol for a scoping review. BMJ Open. 2021 Aug 6;11(8):e047280.
4. Hagopian A, Rao D, Katz A, Sanford S, Barnhart S. Anti-homosexual legislation and HIV-related stigma in African nations: what has been the role of PEPFAR? Glob Health Action. 2017 Jan 5;10(1):1306391.
5. Price MSPH, PhD MA, Rida PhD W, Mwangome MBChB, MSc M, Mutua MBChB, MPH G, Middelkoop MBChB, PhD K, Roux MBChB, MPH S, et al. Identifying At-Risk Populations in Kenya and South Africa: HIV Incidence in Cohorts of Men Who Report Sex With Men, Sex Workers, and Youth. J Acquir Immune Defic Syndr (1988). 2012;
6. Phaswana-Mafuya, Simbayi L, Wabiri N, Cloete A, Ghana AIDS Commission. "The Ghana men's study II: mapping and population size estimation (MPSE) and integrated bio-behavioral surveillance survey (IBBSS) amongst men who have sex with men (MSM) in Ghana." 2020.
7. Ghana AIDS Commission. President's Emergency Plan for AIDS Relief, US Centers for Disease Control, University of California San Francisco. The Ghana Men's Study: Integrated biological-behavioral surveillance surveys and population size estimation among men who have sex with men in Ghana. . Accra, Ghana; 2013.
8. Ghana National AIDS Control Programme. National HIV prevalence & AIDS estimates report, 2012–2013. . Accra, Ghana; 2016.
9. Abubakari GMR, Nelson LRE, Ogunbajo A, Boakye F, Appiah P, Odhiambo A, et al. Implementation and evaluation of a culturally grounded group-based HIV prevention programme for men who have sex with men in Ghana. Glob Public Health. 2021;16(7).
10. Abubakari GMR, Turner DA, Ni Z, Conserve DF, Dada D, Otchere A, et al. Community-Based Interventions as Opportunities to Increase HIV Self-Testing and

Linkage to Care Among Men Who Have Sex With Men – Lessons From Ghana, West Africa. Front Public Health. 2021;9.

11. Abubakari GM, Owusu-Dampare F, Ogunbajo A, Gyasi J, Adu M, Appiah P, et al. HIV Education, Empathy, and Empowerment (HIVE3): A Peer Support Intervention for Reducing Intersectional Stigma as a Barrier to HIV Testing among Men Who Have Sex with Men in Ghana. Int J Environ Res Public Health. 2021 Dec 12;18(24):13103.

12. Kushwaha S, Lalani Y, Maina G, Ogunbajo A, Wilton L, Agyarko-Poku T, et al. "but the moment they find out that you are MSM ⋯": a qualitative investigation of HIV prevention experiences among men who have sex with men (MSM) in Ghana's health care system. BMC Public Health. 2017;17(1).

13. Ogunbajo A, Oke T, Okanlawon K, Abubakari GM, Oginni O. Religiosity and Conversion Therapy is Associated with Psychosocial Health Problems among Sexual Minority Men (SMM) in Nigeria. J Relig Health. 2022;61(4).

14. Gyamerah AO, Taylor KD, Atuahene K, Anarfi JK, Fletcher M, Raymond HF, et al. Stigma, discrimination, violence, and HIV testing among men who have sex with men in four major cities in Ghana. AIDS Care. 2020 Aug 2;32(8):1036–44.

15. Darteh EKM, Kumi-Kyereme A, Awusabo-Asare K. Perception of risk of HIV among adolescents' living in an Urban Slum in Ghana. Afr J Reprod Health. 2016;20(1).

16. Musinguzi G, Bastiaens H, Matovu JKB, Nuwaha F, Mujisha G, Kiguli J, et al. Barriers to condom use among high risk men who have sex with men in Uganda: A qualitative study. PLoS One. 2015;10(7).

17. Engstrom R, Pavelesku D, Tanaka T, Wambile A. Mapping poverty and slums using multiple methodologies in Accra, Ghana. In: 2019 Joint Urban Remote Sensing Event, JURSE 2019. 2019.

18. Esantsi SF, Onyango F, Asare GQ, Kuffour E, Tapsoba P, Birungi H, et al. Understanding the reproductive health needs of adolescents in selected slums in Ghana: a public health assessment. Ghana; 2015.

19. Kabiru CW, Beguy D, Crichton J, Zulu EM. HIV/AIDS among youth in urban informal (slum) settlements in Kenya: What are the correlates of and motivations for HIV testing? BMC Public Health. 2011 Dec 3;11(1):685.

20. J. Madise N, Ziraba AK, Inungu J, Khamadi SA, Ezeh A, Zulu EM, et al. Are slum dwellers at heightened risk of HIV infection than other urban residents? Evidence from population-based HIV prevalence surveys in Kenya. Health Place. 2012;18(5).

21. Amoako C, Cobbinah PB. Slum improvement in the Kumasi metropolis,Ghana - a review of approaches and results. Journal of Sustainable Development in Africa. 2011;13(8).

22. Snyder RE, Boone CE, Cardoso CAA, Aguiar-Alves F, Neves FPG, Riley LW. Zika: A scourge in urban slums. PLoS Negl Trop Dis. 2017 Mar 23;11(3):e0005287.

23. Teixeira PJ, Marques MM, Silva MN, Brunet J, Duda JL, Haerens L, et al. A classification of motivation and behavior change techniques used in self-determination theory-based interventions in health contexts. Motiv Sci. 2020;6(4).

24. Ryan RM, Deci EL. Intrinsic and extrinsic motivation from a self-determination theory perspective: Definitions, theory, practices, and future directions. Contemp Educ Psychol. 2020;61.

25. Nelson LE, Wilton L, Agyarko-Poku T, Zhang N, Aluoch M, Thach CT, et al. The Association of HIV Stigma and HIV/STD Knowledge With Sexual Risk Behaviors Among Adolescent and Adult Men Who Have Sex With Men in Ghana, West Africa. Res Nurs Health. 2015 Jun;38(3):194–206.

26. Nelson LRE, Wilton L, Agyarko-Poku T, Zhang N, Aluoch M, Thach CT, et al. The Association of HIV Stigma and HIV/STD Knowledge With Sexual Risk Behaviors Among Adolescent and Adult Men Who Have Sex With Men in Ghana, West Africa. Res Nurs Health. 2015;38(3).

27. Faust L, Yaya S. The effect of HIV educational interventions on HIV-related knowledge, condom use, and HIV incidence in sub-Saharan Africa: A systematic review and meta-analysis. Vol. 18, BMC Public Health. 2018.

28. Mbeda C, Ogendo A, Lando R, Schnabel D, Gust DA, Guo X, et al. Healthcare-related stigma among men who have sex with men and transgender women in sub-Saharan Africa participating in HIV Prevention Trials Network (HPTN) 075 study. AIDS Care - Psychological and Socio-Medical Aspects of AIDS/HIV. 2020;32(8).

29. Luvuno ZP, Mchunu G, Ncama B, Ngidi H, Mashamba-Thompson T. Evidence of interventions for improving healthcare access for lesbian, gay, bisexual and transgender people in South Africa: A scoping review. Afr J Prim Health Care Fam Med. 2019;11(1).

30. Keuroghlian AS, Mujugira A, Mayer KH. Healthcare worker training to improve quality of care for sexual and gender minority people in sub-Saharan Africa: learning from efforts in Uganda. Vol. 24, Journal of the International AIDS Society. 2021.

31. Nelson LRE, Nyblade L, Torpey K, Logie CH, Qian HZ, Manu A, et al. Multi-level intersectional stigma reduction intervention to increase HIV testing among men who have sex with men in Ghana: Protocol for a cluster randomized controlled trial. PLoS One. 2021;16(11 November).

32. Haruna U. Stirring the Hornet's Nest: a Study of Student's Awareness, Perception and Tolerance of Homosexuality in a Ghanaian University. Journal of Sociological Research. 2015;6(1).

33. Adua JY. Religion and Homosexuality in Ghana: Assessing the Factors Constraining the Legalisation of Homosexuality in Ghana: A Study of the Klottey Korle Sub-Metropolitan Area. of Accra. University of Ghana; 2018.

34. Ogunbajo A, Kershaw T, Kushwaha S, Boakye F, Wallace-Atiapah ND, Nelson LE. Barriers, Motivators, and Facilitators to Engagement in HIV Care Among HIV-Infected Ghanaian Men Who have Sex with Men (MSM). AIDS Behav. 2018 Mar 26;22(3):829–39.

35. Girault P, Green K, Clement NF, Rahman YAA, Adams B, Wambugu S. Piloting a Social Networks Strategy to Increase HIV Testing and Counseling Among Men Who Have Sex with Men in Greater Accra and Ashanti Region, Ghana. AIDS Behav. 2015;19(11).

36. Ghana AIDS Commission. Ghana AIDS Commission (2016). National HIV/AIDS strategic plan (2016 – 2020).http://www.ghanaids.gov.gh/gac1/pubs/COMPREHENSIVE%20NSP%202016-2020.pdf [Ref list] . 2016.

37. Ali H, Amoyaw F, Baden D, Durand L, Bronson M, Kim A, et al. Ghana's HIV epidemic and PEPFAR's contribution towards epidemic control. Ghana Med J. 2019 Mar 10;53(1):59.

38. Abubakari GM, Smith MDR, Boyd DT, Raquel Ramos S, Johnson C, Benavides JL, et al. Assessing Different Types of HIV Communication and Sociocultural Factors on Perceived HIV Stigma and Testing among a National Sample of Youth and Young Adults. Int J Environ Res Public Health. 2022;19(2).

39. Boyd DT, Waller B, Quinn CR. Reimaging an AIDS free generation: Examining youth and young adults' personal agency and its association with HIV testing. Prev Med Rep. 2021;22.

40. Abubakari GM, Turner D, Nelson LE, Odhiambo AJ, Boakye F, Manu A, et al. An application of the ADAPT-ITT model to an evidence-based behavioral HIV prevention intervention for men who have sex with men in Ghana. International Health Trends and Perspectives. 2021;1(1).

41. Boyd D, Lea CH, Gilbert KL, Butler-Barnes ST. Sexual health conversations: Predicting the odds of HIV testing among black youth and young adults. Child Youth Serv Rev. 2018;90.

42. Nelson LE, Ogunbajo A, Abu-Ba'are GR, Conserve DF, Wilton L, Ndenkeh JJ, et al. Using the Implementation Research Logic Model as a Lens to View Experiences of

Implementing HIV Prevention and Care Interventions with Adolescent Sexual Minority Men—A Global Perspective. AIDS Behav. 2022 Aug 10;

43. Logie CH, Newman PA, Chakrapani V, Shunmugam M. Adapting the minority stress model: Associations between gender non-conformity stigma, HIV-related stigma and depression among men who have sex with men in South India. Soc Sci Med. 2012;74(8).

44. Saalim K, Amu-Adu P, Amoh-Otu RP, Akrong R, Abu-Ba'are GR, Stockton MA, et al. Multi-level manifestations of sexual stigma among men with same-gender sexual experience in Ghana. BMC Public Health. 2023 Jan 24;23(1):166.

45. Estem KS, Catania J, Klausner JD. HIV Self-Testing: a Review of Current Implementation and Fidelity. Vol. 13, Current HIV/AIDS Reports. 2016.

46. Wilton L, Herbst JH, Coury-Doniger P, Painter TM, English G, Alvarez ME, et al. Efficacy of an HIV/STI prevention intervention for black men who have sex with men: findings from the many men, many voices (3MV) project. AIDS Behav. 2009;13(3).
