# Supplementary material for: Qualitative description of interpersonal HIV stigma and motivations for HIV testing among gays, bisexuals, and men who have sex with men in Ghana’s slums—BSGH-005
Source: PLoS One. 2024 Jun 5;19(6):e0289905. doi: 10.1371/journal.pone.0289905 (PMC11152275; doi:10.1371/journal.pone.0289905)
Supplement: Attachment — Submitted filename: Plos One Review_300324.docx [file pone.0289905.s001.docx]

**General Observations**

This manuscript is about HIV stigma and factors influencing HIV testing among GBMSM in slums of Accra, Ghana. The study employed qualitative data collection methods and reports several findings. On HIV stigma, the study reports that people stay away of avoids GBMSM who are living with HIV and that HIV testing is feared in this sub-population. Factors influencing uptake of HIV testing among GBMSM include perceived vulnerability to HIV, Sexual health decision making, and positive messages about HIV testing. The paper is novel as it addressed literature gaps pertaining to our understanding about the interface between GBMSM and HIV in urban slums. The

“While the study appears to be sound, most of the sections especially in the methods and results sections appear to be underdeveloped. For the methods section, I advise the authors to use the COnsolidated criteria for REporting Qualitative research (COREQ) checklist. For the results section, I suggest authors to provide a discussion or interpretation of the quotes that they have provided. For the Results section, some findings are presented in two or three sentences which suggest that the analysis process was incomplete. I advise the authors to also provide a description or interpretation of the quotes. In some case, language is unclear, making it difficult to follow. I advise the authors review the manuscript to improve the flow and readability of the text. I have provided specific comments below.

**Introduction**

Page 3, Paragraph 1, Sentence 2: Kindly specify the context in which these pragmatic efforts occur e.g., despite pragmatic efforts in SSA... or in Ghana....

Page 3, Paragraph 2, Sentence 1: Please indicate '... compared to that of the general population ... '

Page 3, Paragraph 2, Sentence 2: Please avoid repeating the same word in a sentence. Here, GBMSM has already been used. please use the following phrase '... in this group...'

Page 3, Paragraph 2, Sentence 2: Here, only individual, and environmental barriers have been mentioned. However, in subsequent sentences, there is personal, environmental, and interpersonal barriers. Kindly include interpersonal barriers here.

Page 3, Paragraph 2, Sentence 3: Is 'individual level' the same as 'personal level'? Please be consistent in the use of the words.

Page 3, Paragraph 3, Sentence 1: Kindly provide references for these limited studies.

Page 3, Paragraph 3, Sentence 2: This sentence is not coherent with the preceding sentence. There is a disconnect between the first and the second sentences. Please revisit the sentence.

Page 3, Paragraph 3, Sentence 5: Please provide the year when these results were published. For example, in Kenya in 2024, HIV rates among slum...

Page 4, Paragraph 2, Sentence 1: Reference is needed here.

Page 4, Paragraph 2, Sentence 2: This sentence will sound better if written: 'Communities with increased HIV prevalence and risk also have poorly resourced infrastructure and health facilities which contributes to poor health outcomes’.

Page 4, Paragraph 2, Sentence 3: Delete ' where GBMSM were sampled for the study'. This information should be provided in the methodology section.

Page 4, Paragraph 3, Sentence 1: It is important to clarify the type of stigma being referred to here. Is this stigma broadly, self-stigma or other forms of stigma.

Page 4, Paragraph 3, Sentence 3: Excellent to have includes a theoretical framework.

Page 4, Paragraph 3, Sentence 4: Can the theory also explain the HIV stigma dimension that also underpin this study? The theory should be used for all dimension of your study.

Page 4, Paragraph 3, Sentence 6: I think that these dimensions should be part of the discussion section. In the introduction, just describe what the theory is and what are its components. A description of each component is also important in the introductions section.

**Methodology**

Start this section with the study design. Kindly use the COREQ checklist to guide the content and structure of your methods section.

Page 5, Paragraph 2, Sentence 2: If you are talking about partner organisations, it would be necessary to also mention the main organisation that implemented this work within the methods section.

Page 5, Paragraph 2, Sentence 4: This sentence will read better if it begins like this.

Although we originally intended to purposively include 19 participants in this study, we reached saturation of information after the eighth interview.

Page 5, Paragraph 2, Sentence 4: How did you determine that saturation of information has been attained? Kindly provide examples of elements that you saw in the data to be confident that you reached information saturation.

Page 5, Paragraph 2, Sentence 3: Full stop is needed at the end of the sentence.

Page 5, Paragraph 3, Sentence 2: Kindly use past tense

Page 6, Paragraph 2, Sentence 3: Provide some background information on the qualifications of research assistants to do this work with this group of study participants. If these research assistants are part of the authors, kindly use their initials. E.g., Experienced graduate researchers (GRA and EYZ) conducted the interviews for this study.

Page 6, Paragraph 2, Sentence 5: Write interviews instead of conversations.

Page 6, Paragraph 3, Sentence 1: Please provide the number of days that this training was done and whether, protocol training was part of this training.

Page 7, Paragraph 1, Sentence 1: How was data processing done. Transcription and translation considering that some participants were interviewed in Twi.

Page 7, Paragraph 1, Sentence 1: Are these crucial points or codes?

**Results**

I would suggest that the authors should use the word 'Findings' instead of 'Results' which is mostly used in quantitative studies.

Page 7, Paragraph 3, Sentence 3: It would be good to include age range of the participant and their current occupation.

Page 7, Paragraph 3, Sentence 2: This sentence should be phrased 'Five participants accomplished tertiary education, six concluded senior high school while one didn't complete Junior High school education.

Page 8, Paragraph 1, Sentence 1: These groups the way this statement is written suggest that they were generated deductively based on researcher’s priori thoughts and not inductively. Kindly confirm if this observation is accurate and include a statement that these categories were deductively generated.

Page 8, Paragraph 1, Sentence 1: Are the main groups themes? Please check the language used to be consistent with language used in qualitative research methodologies.

Page 8, Paragraph 1, Sentence 2: Are the sub-categories sub-themes?

Page 8, Paragraph 1, Sentence 2: Are the sub-categories sub-themes?

Page 8, Paragraph 2, Sentence 1: This sentence will read better if written: Our findings show that study participants feared HIV broadly and avoided being associated with PLHIV in their community. They also lacked proper information about how HIV is transmitted from one person to another.

Page 8, Paragraph 3, Sentence 1: I think that this section is underdeveloped. It needs more information. Include information about why they fear HIV positive test results. In the second quote, the participant mentions some elements of this fear. People usually are afraid of being bonded to lifetime treatment. There may be issues of HIV stigma among other factors.

Page 8, Paragraph 3, Sentence 2: This sentence is not clear and does not link well with previous sentences.

Page 9, Quotes: Some analysis or discussion of what these quotes are portraying is important. For example: The first quote demonstrates how lack of knowledge about HIV transmission precipitates fear and subsequently HIV stigma.

Page 9, Quote 2: I have noted that the authors only use the word 'GBMSM participant' for all quotes. I think that it will be good to add more information such as participant ID number and age so that we are sure that these quotes came from several participants and not just a few of them.

Page 9, Subtitle - Sexual Health Decision Making 1: I suggest that you should revise the subtitle: Knowing ones HIV status

Page 10, Quote 1: The word 'if' should begin with a capital letter.

**Discussion section**

Page 11, Paragraph 1; Sentence 1: This sentence is not clear. Kindly revise it.

Page 11, Paragraph 1, Sentence 4: This sentence should include the word vulnerability: 'The need to test was driven by factors such as perceived vulnerability...

Page 11, Paragraph 1, Last Sentence: The sentence is missing something. Do you mean that these findings highlight the need for interventions to ...

Page 11, Paragraph 2, Sentence 2: A phrase '...among some participants...' should be either among individuals or among people or among GBMSM.

Page 11; Paragraph 2, Sentence 4: I think that you should delete 'Such interventions remain essential considering that the participants did not only have negative misconceptions about HIV.' A revised sentence should read.

The present study findings of participants projecting stigma towards their peers living with HIV by avoiding them, refusing them sex, and labelling them as sexually promiscuous was also reflected in our previous findings among other GBMSM.

Page 12, Paragraph 1, Sentence 1: I am not clear about a phrase '...opportunities for leveraging motivated GBMSM...'

Page 12, Paragraph 1, Sentence 2: A phrase '...encouraged them to test to protect themselves and their partners.' is not clear. HIV testing does not directly provide protection from HIV infection, but knowledge of HIV status allows individuals to embrace HIV prevention or initiate treatment which also serves as prevention. I suggest that this sentence should end with '...encouraged them to test.'

Page 12, Paragraph 2; Sentence 3: please delete 'also’.

Page 12, Paragraph 2; Sentence 4: delete the word 'to’.

Page 12, Paragraph 2; Sentence 5: delete the word 'also’.

Page 12, Paragraph 2; Sentence 5: I am not sure about the meaning of '...health sexual health behaviour...'

Page 12, Paragraph 3; Sentence 1: I suggest that you should delete a phrase 'To emphasize further...' in this sentence or mention the point that is being emphasised.

Start this sentence as follows: Providing information on how HIV is transmitted...'

Page 13, Paragraph 1; Sentence 2: delete the word 'the'.

Page 13, Paragraph 2; Sentence 1: End this sentence as follows: '...when interpreting these study findings.

Page 12, Paragraph 2; Sentence 2: I think that qualitative designs are important for their depth within the context where the study has been done and not their generalisability or applicability in contexts elsewhere. I think that this limitation does not apply to this study.

Page 12, Paragraph 2; Sentence 4: This also is not a methodological limitation for qualitative studies.
