# Supplementary material for: Qualitative description of interpersonal HIV stigma and motivations for HIV testing among gays, bisexuals, and men who have sex with men in Ghana’s slums—BSGH-005
Source: PLoS One. 2024 Jun 5;19(6):e0289905. doi: 10.1371/journal.pone.0289905 (PMC11152275; doi:10.1371/journal.pone.0289905)
Supplement: Attachment — Submitted filename: Response to Reviewers..doc [file pone.0289905.s003.doc]

**Response for manuscript** **“Qualitative description of interpersonal** **HIV stigma and motivations for HIV testing among gays, bisexuals, and men who have sex with men in Ghana's slums - BSGH-005”**

Dear reviewers,

We have addressed the following changes mentioned by the reviewers and highlighted these in yellow under each recommended statement. Additionally, we have reviewed the entire manuscript to fix grammatical errors in all sections.

**Introduction**

Page 3, Paragraph 1, Sentence 2: Kindly specify the context in which these pragmatic efforts occur e.g., despite pragmatic efforts in SSA... or in Ghana.... “in SSA” inserted.

Page 3, Paragraph 2, Sentence 1: Please indicate '... compared to that of the general population ... '“nation’s” changed to “that of the general population”

Page 3, Paragraph 2, Sentence 2: Please avoid repeating the same word in a sentence. Here, GBMSM has already been used. please use the following phrase '... in this group...' GBMSM changed to “in this group”

Page 3, Paragraph 2, Sentence 2: Here, only individual, and environmental barriers have been mentioned. However, in subsequent sentences, there is personal, environmental, and interpersonal barriers. Kindly include interpersonal barriers here. “interpersonal,” inserted.

Page 3, Paragraph 2, Sentence 3: Is 'individual level' the same as 'personal level'? Please be consistent in the use of the words. “Personal” changed to “individual”

Page 3, Paragraph 3, Sentence 1: Kindly provide references for these limited studies.

Page 3, Paragraph 3, Sentence 2: This sentence is not coherent with the preceding sentence. There is a disconnect between the first and the second sentences. Please revisit the sentence. Sentence changed to “However, studies have highlighted the association of low HIV knowledge and low HIV testing to persons with low socioeconomic status”.

Page 3, Paragraph 3, Sentence 5: Please provide the year when these results were published. For example, in Kenya in 2024, HIV rates among slum... Sentence changed to “In 2012, HIV rates among slum residents in Kenya were notably higher at 12%, compared to 5% among non-slum urban residents and 6% among rural residents”.

Page 4, Paragraph 2, Sentence 1: Reference is needed here. We have added the references to this sentence (18,21,22).

Page 4, Paragraph 2, Sentence 2: This sentence will sound better if written: 'Communities with increased HIV prevalence and risk also have poorly resourced infrastructure and health facilities which contributes to poor health outcomes’. Sentence changed to “Communities with increased HIV prevalence and risk also have poorly resourced infrastructure and health facilities which contributes to poor health outcomes”.

Page 4, Paragraph 2, Sentence 3: Delete ' where GBMSM were sampled for the study'. This information should be provided in the methodology section. This sentence has been deleted.

Page 4, Paragraph 3, Sentence 1: It is important to clarify the type of stigma being referred to here. Is this stigma broadly, self-stigma or other forms of stigma. To clarify, “manifestation” inserted

Page 4, Paragraph 3, Sentence 3: Excellent to have includes a theoretical framework. Thank you

Page 4, Paragraph 3, Sentence 4: Can the theory also explain the HIV stigma dimension that also underpin this study? The theory should be used for all dimension of your study. We have included the HIV stigma dimension to the theory section of the manuscript.

Page 4, Paragraph 3, Sentence 6: I think that these dimensions should be part of the discussion section. In the introduction, just describe what the theory is and what are its components. A description of each component is also important in the introductions section. We agree this review will give an extensive explanation of how GBMSM experience HIV testing and associated motivations. We have included this in the discussion section and how this relates to some of our findings.

**Methodology**

Start this section with the study design. Kindly use the COREQ checklist to guide the content and structure of your methods section. We have started this section with the study design as indicated. A study setting section has also been included in the method section.

Page 5, Paragraph 2, Sentence 2: If you are talking about partner organisations, it would be necessary to also mention the main organisation that implemented this work within the methods section. Due to the prevailing tension within the region and country where this study was conducted, all organizations based in Ghana that participated in the study have been intentionally anonymized.

Page 5, Paragraph 2, Sentence 4: This sentence will read better if it begins like this: Although we originally intended to purposively include 19 participants in this study, we reached saturation of information after the eighth interview. . “…purposively include 19 participants in this study,..” inserted.

Page 5, Paragraph 2, Sentence 4: How did you determine that saturation of information has been attained? Kindly provide examples of elements that you saw in the data to be confident that you reached information saturation. We inserted “Although we originally intended to purposively include 19 participants in this study, we reached saturation of information after the eight interview. When responses from study participants were found to be consistent by the research assistants, an additional four transcripts were included to ensure complete information saturation, bringing the total number of transcripts to twelve.”

Page 5, Paragraph 2, Sentence 3: Full stop is needed at the end of the sentence. “Full stop” inserted.

Page 5, Paragraph 3, Sentence 2: Kindly use past tense. “Must have” inserted.

Page 6, Paragraph 2, Sentence 3: Provide some background information on the qualifications of research assistants to do this work with this group of study participants. If these research assistants are part of the authors, kindly use their initials. E.g., Experienced researchers (EYZ, OWS) conducted the interviews for this study. We have inserted the all research assistants (EYZ, OWS) engaged in data collection have had prior and extensive research training from working with these populations in the past. We have also utilized the expertise of these researchers in our past studies, with similar populations (21,22,33,34), hence, the familiarity and experience engaging our participants. For the security purposes, partner organizations in Ghana that were involved in locating and recruiting have been deliberately left out of this study.

Page 6, Paragraph 2, Sentence 5: Write interviews instead of conversations. “conversations” changed to “interviews”.

Page 6, Paragraph 3, Sentence 1: Please provide the number of days that this training was done and whether, protocol training was part of this training. Data collection lasted for 10 days, all interviewers have had prior research experience (21,22,33,34), human subject training and certification from the University of Rochester.

Page 7, Paragraph 1, Sentence 1: How was data processing done. Transcription and translation considering that some participants were interviewed in Twi. Data collected in *Twi* were translated and transcribed into the English Language.

Page 7, Paragraph 1, Sentence 1: Are these crucial points or codes? We have changed this to statements to show important points made by GBMSM during interviews.

**Results**

We've included several write-ups into the findings section, to effectively explain the quotes that convey GBMSM responses. By clarifying these findings and providing an extensive explanation, we aim to enhance understanding in the discussion section.

I would suggest that the authors should use the word 'Findings' instead of 'Results' which is mostly used in quantitative studies. “Results” changed to “Findings”

Page 7, Paragraph 3, Sentence 3: It would be good to include age range of the participant and their current occupation. Age range data were not recorded. However, all participants in the study were 18 years and above.

Page 7, Paragraph 3, Sentence 2: This sentence should be phrased 'Five participants accomplished tertiary education, six concluded senior high school while one didn't complete Junior High school education. Sentence was changed to “Five participants accomplished tertiary education, six concluded senior high school while one didn't complete Junior High school education”.

Page 8, Paragraph 1, Sentence 1: These groups the way this statement is written suggest that they were generated deductively based on researcher’s priori thoughts and not inductively. Kindly confirm if this observation is accurate and include a statement that these categories were deductively generated. All categories and subcategories were generated deductively from the emerging data collected and analysed by the researchers. We have rewritten this sentence by indicating “Findings from the data collected and analysed revealed two categories; 1) HIV stigma and 2) Motivations for HIV testing.”

Page 8, Paragraph 1, Sentence 1: Are the main groups themes? Please check the language used to be consistent with language used in qualitative research methodologies. These are categories and subcategories. We have addressed this review and made the necessary changes in the manuscript.

Page 8, Paragraph 1, Sentence 2: Are the sub-categories sub-themes? We have rewritten the paragraph to address this review. “Findings from the data collected and analysed revealed two categories; 1) HIV stigma and 2) Motivations for HIV testing. Under HIV stigma, we identified two subcategories, avoidance of GBMSM living with HIV, and fear of testing positive for HIV. Under the category motivations for HIV testing, we identified three subcategories; HIV vulnerability, sexual health decision-making, and positive messaging about HIV.”

Page 8, Paragraph 1, Sentence 2: Are the sub-categories sub-themes? We have rewritten the paragraph to address this review. “Findings from the data collected and analysed revealed two categories; 1) HIV stigma and 2) Motivations for HIV testing. Under HIV stigma, we identified two subcategories, avoidance of GBMSM living with HIV, and fear of testing positive for HIV. Under the category motivations for HIV testing, we identified three subcategories; HIV vulnerability, sexual health decision-making, and positive messaging about HIV.”

Page 8, Paragraph 2, Sentence 1: This sentence will read better if written: Our findings show that study participants feared HIV broadly and avoided being associated with PLHIV in their community. They also lacked proper information about how HIV is transmitted from one person to another. The sentence was changed to “Our findings show that study participants largely feared HIV and avoided being associated with PLHIV in their community. They also lacked proper information about how HIV is transmitted from one person to another”.

Page 8, Paragraph 3, Sentence 1: I think that this section is underdeveloped. It needs more information. Include information about why they fear HIV positive test results. In the second quote, the participant mentions some elements of this fear. People usually are afraid of being bonded to lifetime treatment. There may be issues of HIV stigma among other factors. Paragraph was changed to “Some participants expressed experience with testing for HIV. However, some shared that they are usually scared of testing for HIV due to the fear of testing positive. Some participants said they had to encourage themselves to go for HIV testing, and struggle to remain optimistic for a negative test result, suggesting manifestations of HIV stigma, and low knowledge on HIV treatment options.”

Page 8, Paragraph 3, Sentence 2: This sentence is not clear and does not link well with previous sentences. Paragraph was changed to “Some participants expressed experience with testing for HIV. However, some shared that they are usually scared of testing for HIV due to the fear of testing positive. Some participants said they had to encourage themselves to go for HIV testing, and struggle to remain optimistic for a negative test result, suggesting manifestations of HIV stigma, and low knowledge on HIV transmission and treatment options.”

Page 9, Quotes: Some analysis or discussion of what these quotes are portraying is important. For example: The first quote demonstrates how lack of knowledge about HIV transmission precipitates fear and subsequently HIV stigma. Paragraph was changed to “Some participants expressed experience with testing for HIV. However, some shared that they are usually scared of testing for HIV due to the fear of testing positive. Some participants said they had to encourage themselves to go for HIV testing, and struggle to remain optimistic for a negative test result, suggesting manifestations of HIV stigma, and low knowledge on HIV transmission and treatment options.”

Page 9, Quote 2: I have noted that the authors only use the word 'GBMSM participant' for all quotes. I think that it will be good to add more information such as participant ID number and age so that we are sure that these quotes came from several participants and not just a few of them. We have accepted the importance of this review and included the initials of unique codes assigned to each participant to separate the responses of GBMSM.

Page 9, Subtitle - Sexual Health Decision Making 1: I suggest that you should revise the subtitle: Knowing ones HIV status. Subtitle changed to “Knowing one’s HIV status”

Page 10, Quote 1: The word 'if' should begin with a capital letter. 'if' changed to “If”(capital letter).

**Discussion section**

Page 11, Paragraph 1; Sentence 1: This sentence is not clear. Kindly revise it. We have rewritten this sentence to sound “Despite the heightened stigma, elevated vulnerability to HIV, and low rates of HIV testing among GBMSM and residents of slums, there is a lack of research exploring HIV testing among GBMSM, particularly within slum communities in Ghana (10,38–40)”

Page 11, Paragraph 1, Sentence 4: This sentence should include the word vulnerability: 'The need to test was driven by factors such as perceived vulnerability... Sentence was changed to “The need to test was driven by factors such as perceived HIV vulnerability, need to know one’s HIV status for sexual health reasons, and positive messaging about HIV-informed motivations for HIV Testing”.

Page 11, Paragraph 1, Last Sentence: The sentence is missing something. Do you mean that these findings highlight the need for interventions to ...we accept this reveal and have rewritten this sentence “These findings emphasize the necessity for interventions that enhance HIV awareness and capitalize on existing motivation to enhance HIV testing rates among GBMSM residing in slum areas”.

Page 11, Paragraph 2, Sentence 2: A phrase '...among some participants...' should be either among individuals or among people or among GBMSM. “...some participants...” changed to “GBMSM”.

Page 11; Paragraph 2, Sentence 4: I think that you should delete 'Such interventions remain essential considering that the participants did not only have negative misconceptions about HIV.' A revised sentence should read. Sentence was changed to “The present study findings of participants projecting stigma towards their peers living with HIV by avoiding them, refusing them sex, and labelling them as sexually promiscuous was also reflected in our previous findings among other GBMSM”.

Page 12, Paragraph 1, Sentence 1: I am not clear about a phrase '...opportunities for leveraging motivated GBMSM...'' Change leveraging to “encouraging” or remove “leveraging”. Leveraging has been replaced by encouraging.

Page 12, Paragraph 1, Sentence 2: A phrase '...encouraged them to test to protect themselves and their partners.' is not clear. HIV testing does not directly provide protection from HIV infection, but knowledge of HIV status allows individuals to embrace HIV prevention or initiate treatment which also serves as prevention. I suggest that this sentence should end with '...encouraged them to test.'“to protect themselves and their partners” removed.

Page 12, Paragraph 2; Sentence 3: please delete 'also’. 'also’ removed.

Page 12, Paragraph 2; Sentence 4: delete the word 'to’. 'to’ removed.

Page 12, Paragraph 2; Sentence 5: delete the word 'also’. 'also’ removed.

Page 12, Paragraph 2; Sentence 5: I am not sure about the meaning of '...health sexual health behaviour...' We have rewritten “Whereas no previous studies focused on GBMSM in slums, earlier studies among GBMSM in Ghana showed that some GBMSM acknowledged the importance of HIV testing, as it enabled them know their status and make important sexual health choices”.

Page 12, Paragraph 3; Sentence 1: I suggest that you should delete a phrase 'To emphasize further...' in this sentence or mention the point that is being emphasised. Start this sentence as follows: Providing information on how HIV is transmitted...'. “To emphasize further...” removed. “Providing information on how HIV is transmitted” inserted.

Page 13, Paragraph 1; Sentence 2: delete the word 'the'. “the” removed.

Page 13, Paragraph 2; Sentence 1: End this sentence as follows: '...when interpreting these study findings. Sentence changed to “Despite the important findings, research needs to consider the study limitations when interpreting these study findings”.

Page 12, Paragraph 2; Sentence 2: I think that qualitative designs are important for their depth within the context where the study has been done and not their generalisability or applicability in contexts elsewhere. I think that this limitation does not apply to this study. We agree with this review and have corrected this statement to limit generalisability or applicability to the context of Ghana, and slums located in the country. Hence, findings on the state of GBMSM living in

slum communities in this study could be used to suggest the current state in other slums in Ghana, particularly in the area of HIV stigma and testing.

Page 12, Paragraph 2; Sentence 4: This also is not a methodological limitation for qualitative studies. We agree and have remove the sentence.

Sincerely

Osman Wumpini Shamrock PhD, MPhil

School of Nursing, University of Rochester

225 Crittenden Blvd, Rochester, NY, 14642

+1 (607) 761 8189
