# Supplementary material for: Qualitative description of interpersonal HIV stigma and motivations for HIV testing among gays, bisexuals, and men who have sex with men in Ghana’s slums—BSGH-005
Source: PLoS One. 2024 Jun 5;19(6):e0289905. doi: 10.1371/journal.pone.0289905 (PMC11152275; doi:10.1371/journal.pone.0289905)
Supplement: Attachment — Submitted filename: Response to Reviewers (2).doc [file pone.0289905.s004.doc]

**Response for manuscript** **“Qualitative description of interpersonal** **HIV stigma and motivations for HIV testing among gays, bisexuals, and men who have sex with men in Ghana's slums - BSGH-005”**

Dear reviewers/editor,

We have addressed the following changes mentioned by the reviewers/editor. Additionally, we have reviewed the entire manuscript to fix grammatical errors in all sections.

**Methods**

1. Eight interview-was changed to eight interviews.
2. Additionally, the individual must identify as a cisgender man who self-identifies as gay, bisexual, or pansexual or engage in sexual intercourse with another cis-gender man for reasons other than sexual orientation- Has been revised to Additionally, the individual identity had to be a cisgender man who self-identifies as gay, bisexual, or pansexual or engage in sexual intercourse with another cis-gender man for reasons other than sexual orientation.
3. Research assistants (EYZ, OWS) engaged in data collection have had prior and extensive research training from working with these populations in the past- Has been revised to Research assistants (EYZ, OWS) engaged in data collection had prior and extensive research training from working with these populations in the past.
4. We have also utilized the expertise of these researchers in our past studies, with similar populations, hence, the familiarity and experience engaging our participants- Has been revised to We also utilized the expertise of these researchers in our past studies, with similar populations, hence, the familiarity and experience engaging our participants.
5. “Participants”- has been changed to “participants”.

**Results**

6. “Motivation”- has been changed to “motivation”.
7. Under HIV stigma, we identified two subcategories, avoidance of GBMSM living with HIV, and fear of testing positive for HIV- has been changed to Under HIV stigma: avoidance of GBMSM living with HIV, and fear of testing positive for HIV
8. Under the category motivations for HIV testing, we identified three subcategories: HIV vulnerability, sexual health decision-making, and knowing one’s HIV status - has been changed to Under the category motivations for HIV testing: HIV vulnerability, sexual health decision-making, and knowing one’s HIV status.
9. Participant K, mentioned they were scared to be near someone with HIV because they worried, they might get infected - has been changed to Participant K, mentioned they were scared to be near someone because of fear of a potential HIV infection.
10. Some participants knew a lot about how HIV spreads and were ok with being around people who have HIV - has been changed to Some participants were well informed about how HIV is transmitted and were comfortable with being around people who have HIV.

**Discussion**

11. We employ the SDT theory in the context of this study to highlight the psychological needs for autonomy, competence, and relatedness for GBMSM in HIV testing, or how this affects the motivation for testing. - has been changed to The SDT theory was useful in the context of this study to highlight the psychological needs for autonomy, competence, and relatedness for GBMSM in HIV testing, or how this affects the motivation for testing.'
12. The theory also throws more light on HIV stigma and how this impacts autonomy as fears of negative stereotypes impede testing. - has been changed to The theory also improved our understanding of HIV stigma and how it impacts autonomy considering that the fear of negative stereotypes impedes uptake of testing.
13. Testing - has been changed to testing
14. Reference has been provided for “The present study findings of participants projecting stigma towards their peers living with HIV by avoiding them, refusing them sex, and labelling them as sexually promiscuous was also reflected in our previous findings among other GBMSM in Ghana.”

**References**

15. Number (5) reference has been updated to Price MA, Rida W, Mwangome M, Mutua G, Middelkoop K, Roux S, et al. Identifying at-risk populations in kenya and south africa: HIV incidence in cohorts of menwho report sex with men, sex workers, and youth. J Acquir Immune Defic Syndr (1988). 2012;59(2). doi:10.1097/QAI.0b013e31823d8693
16. Added reference: 31. Dakpui HD, Shamrock OW, Aidoo-Frimpong G, Zigah EY, Agbemedu GR, Ahmed A, et al. A qualitative description of HIV testing and healthcare experiences among trans women in Ghanaian urban slums BSGH-011. 2024. doi:https://doi.org/10.21203/rs.3.rs-4390892/v1
17. Added reference: 6. Abu-Ba’are GR, Torpey K, Nelson L, Conserve D, Jeon S, McMahon J, et al. Adaptation and feasibility of Many Men Many Voices (3MV), an HIV prevention intervention to reduce intersectional stigma and increase HIVST among YSMM residing in Ghanaian slums–A clustered pre-post pilot trial protocol. 2024. Available from: doi: 10.21203/rs.3.rs-4313437/v1
18. Added reference: 20. Osman Wumpini Shamrock, Henry Delali Dakpui, George Rudolph Kofi Agbemedu, Donte T Boyd, Kharul Islam, Ibrahim Wunpini Mashoud, et al. “I’m scared of the nurses telling other people I am a transwoman”: Disclosure and nondisclosure of gender identity among trans women in Ghana’s urban slums– BSGH010 [Internet]. 2024 Apr [cited 2024 Apr 19]. Available from: <https://doi.org/10.21203/rs.3.rs-4243840/v1>
19. All other references have been adjusted to fit the journal’s specifications.

Sincerely

Osman Wumpini Shamrock PhD, MPhil

School of Nursing, University of Rochester

225 Crittenden Blvd, Rochester, NY, 14642

+1 (607) 761 8189
